# Physical activity and health-promoting lifestyles in adults with chronic diseases: serial indirect associations through subjective well-being and self-efficacy in multiethnic areas of Yunnan, China

**DOI:** 10.3389/fpubh.2026.1799074

**Published:** 2026-05-29

**Authors:** Hanglin Yu, Zhaozhi Liu, Hansen Li, Haodong Tian, Li Peng

**Affiliations:** 1 School of Physical Education, Southwest University, Chongqing, China; 2 School of Physical Education, Sichuan Agricultural University, Ya'an, China

**Keywords:** ethnic minority, health-promoting lifestyle, physical activity, self-efficacy to manage chronic disease, subjective well-being, Yunnan

## Abstract

**Objective:**

To examine the association between physical activity and health-promoting lifestyle among adults with chronic diseases in multiethnic areas of Yunnan, China, and to assess the serial indirect associations involving subjective well-being and self-efficacy to manage chronic disease.

**Methods:**

A cross-sectional survey was conducted from October 2024 to June 2025 among 1,988 adults with chronic diseases in ethnic minority-concentrated areas of Yunnan Province, China. A multistage stratified site-based sampling framework with quota-based participant recruitment was used. Yunnan Province was stratified into four geographic regions, prefecture-level areas with high concentrations of ethnic minority populations were identified, community-based and medical-institution-based survey sites were selected, and eligible adults were recruited after standardized screening until site quotas were reached. Health-promoting lifestyle, physical activity, subjective well-being, and self-efficacy to manage chronic disease were assessed using the HPLP-IIR, PARS-3, WHO-5, and SEMCD-6, respectively. Descriptive statistics, t tests or ANOVA, Pearson correlation analysis, multivariable regression analysis, and PROCESS Model 6 with 5,000 bootstrap samples were used.

**Results:**

Participants reported low physical activity (10.22 ± 19.92) and below-moderate health-promoting lifestyle (114.94 ± 39.28). Health-promoting lifestyle differed by age, ethnicity, marital status, employment status, and tobacco/alcohol use. Physical activity, subjective well-being, self-efficacy, and health-promoting lifestyle were positively correlated, and physical activity, subjective well-being, and self-efficacy were each positively associated with health-promoting lifestyle in adjusted regression models. The total association between physical activity and health-promoting lifestyle was 0.565, with a total indirect association of 0.174 (30.9%) through subjective well-being and self-efficacy; all indirect association pathways had 95% CIs excluding zero.

**Conclusion:**

Among adults with chronic diseases in multiethnic areas of Yunnan, physical activity was positively associated with health-promoting lifestyle, with significant serial indirect associations involving subjective well-being and self-efficacy to manage chronic disease.

## 1 Introduction

With population aging and the growing burden of chronic non-communicable diseases in China, promoting health-promoting lifestyle (HPL) has become an important public health and clinical nursing concern (1). HPL refers to health-enhancing daily behaviors, including healthy diet, regular physical activity, stress management, positive interpersonal relationships, and active health responsibility (2). It is associated with lower risk of chronic conditions such as cardiovascular disease and diabetes (3), as well as better mental health, well-being, and quality of life (4).

Physical activity (PA) is a core component of HPL. Regular PA is associated with better cardiorespiratory function, metabolic status, and body weight (5, 6), as well as lower depression and anxiety and higher subjective well-being and life satisfaction (7). However, PA levels remain relatively low in some regions of China, especially among individuals with chronic diseases and populations living in multiethnic areas (8). Insufficient PA may be related to weaker physical function and poorer HPL dimensions such as exercise, dietary control, and stress management (9). Yunnan is one of China’s most ethnically diverse provinces, and its ethnic minority-concentrated areas have complex terrain, uneven health service accessibility, and heterogeneous dietary habits, chronic disease knowledge, and lifestyle patterns (10, 11). Adults with chronic diseases in these settings may also face insufficient health education, limited exercise guidance, and weak self-management support (12). Therefore, examining PA and HPL in multiethnic areas of Yunnan has specific public health relevance.

Self-efficacy to manage chronic disease (SEMCD) and subjective well-being (SWB) are important psychological correlates of HPL. SEMCD, derived from Bandura’s social cognitive theory, refers to individuals’ judgment of their ability to organize and execute actions needed to achieve desired goals (13). It is associated with medication adherence, dietary control, and exercise performance (14), and is an important psychological factor in health behavior interventions (15). Previous studies have shown that PA is positively associated with SEMCD, which may be related to healthier lifestyle behaviors (16).

SWB is a subjective evaluation of quality of life and an important indicator of mental health (4). Regular PA is positively associated with SWB, possibly through lower depression and anxiety and more positive affect (17). Individuals with higher SWB may hold more positive health beliefs and be more likely to maintain HPL (18). Previous studies have shown positive correlations between SWB and HPL (19), as well as associations among SWB, SEMCD, and HPL (20, 21).

**Figure 1:**
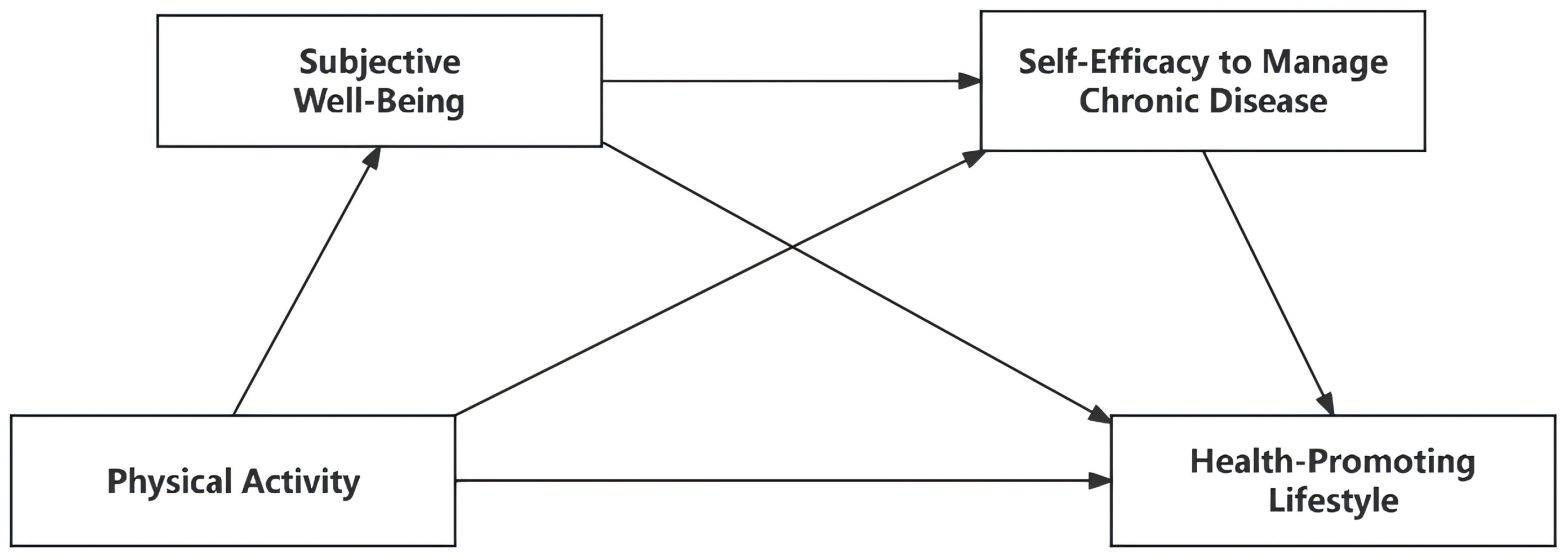
Hypothesized statistical association model.

Most previous studies have examined pairwise relationships among PA, SWB, SEMCD, and HPL (16, 17), while evidence on their joint association pattern remains limited, especially among adults with chronic diseases in multiethnic areas of China (22). This study therefore examined the association between PA and HPL in adults with chronic diseases from multiethnic areas of Yunnan Province and assessed whether SWB and SEMCD were involved in this association in a serial indirect association model. The hypothesized statistical association model is shown in Figure 1.

## 2 Methods

### 2.1 Participants

**Figure 2:**
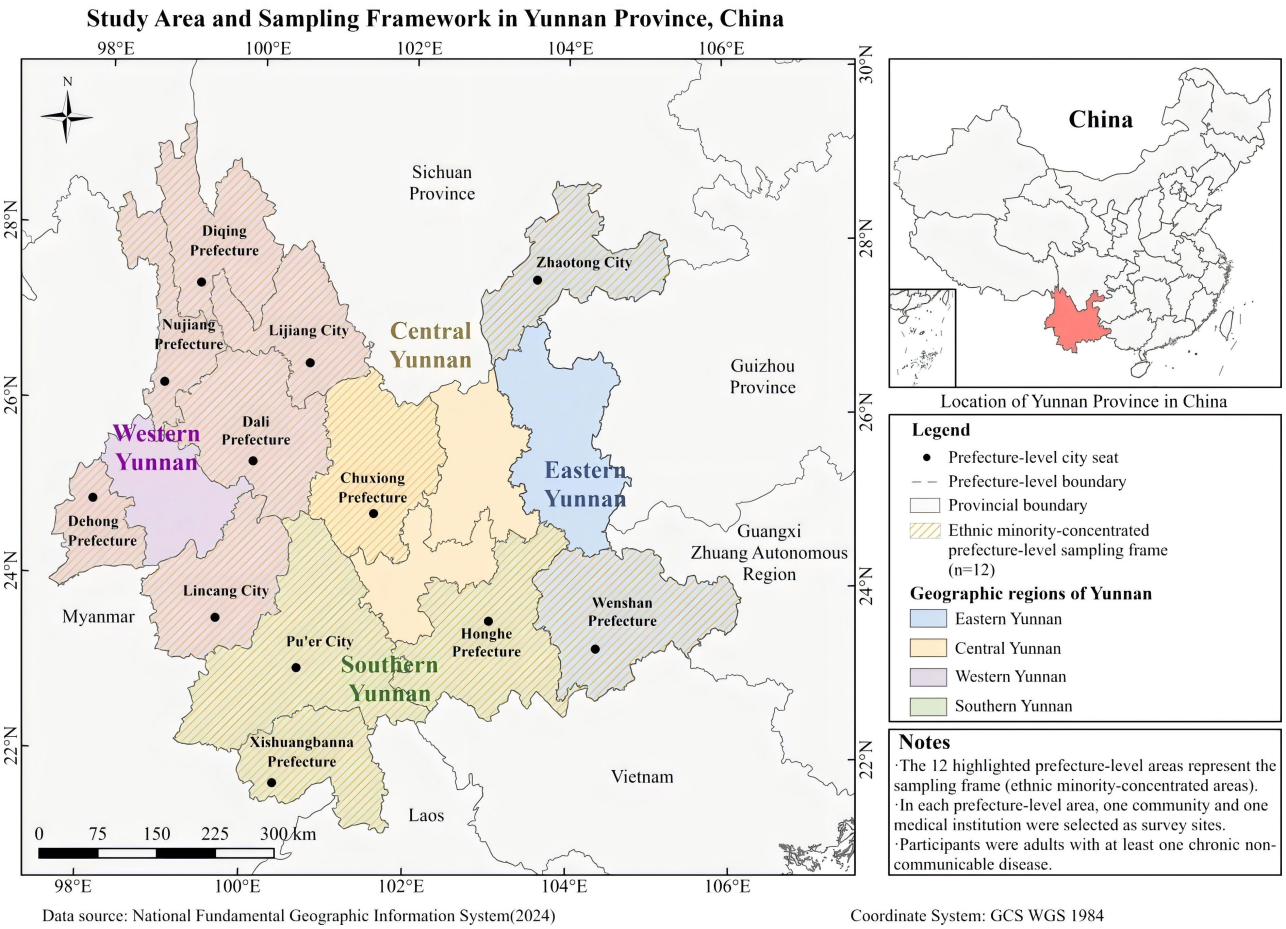
Study area and prefecture-level sampling framework in Yunnan Province, China. The highlighted prefecture-level areas represent the ethnic minority-concentrated sampling frame used in this study. These areas included Chuxiong Prefecture, Zhaotong City, Wenshan Prefecture, Honghe Prefecture, Pu’er City, Xishuangbanna Prefecture, Dali Prefecture, Lijiang City, Nujiang Prefecture, Diqing Prefecture, Dehong Prefecture, and Lincang City. Within these areas, community-based and medical-institution-based survey sites were selected, and eligible adults with chronic non-communicable diseases were recruited after standardized screening.

This cross-sectional study was conducted from October 2024 to June 2025 in ethnic minority-concentrated areas of Yunnan Province, China. A multistage stratified site-based sampling framework with quota-based participant recruitment was used. Yunnan Province was first stratified into central, eastern, southern, and western regions. Prefecture-level areas with high concentrations of ethnic minority populations were then identified as the sampling frame, including Chuxiong Prefecture, Zhaotong City, Wenshan Prefecture, Honghe Prefecture, Pu’er City, Xishuangbanna Prefecture, Dali Prefecture, Lijiang City, Nujiang Prefecture, Diqing Prefecture, Dehong Prefecture, and Lincang City. Community-based and medical-institution-based survey sites were selected within these areas. At each site, potentially eligible adults were screened according to predefined criteria and invited to participate voluntarily after a standardized study introduction until the allocated site quota was reached. Randomization was applied at the geographic and site-selection levels, while individual recruitment was based on eligibility screening, voluntary participation, and quota completion. Therefore, this procedure should not be interpreted as full probability-based random sampling of individuals. The study area and prefecture-level sampling framework are shown in Figure 2. The target population was selected because Yunnan’s ethnic diversity and contextual heterogeneity provide an appropriate setting for examining the PA–SWB–SEMCD–HPL association pattern, and adults with chronic diseases are a priority population for health promotion.

Before data collection, all field investigators received standardized training on questionnaire explanation, respondent communication, and survey administration. During data collection, completed questionnaires were checked daily, and identified errors were corrected promptly. Data were collected through a Chinese online survey platform,^1^ with IP restrictions used to prevent duplicate responses.

Inclusion criteria were as follows: (1) age ≥18 years; (2) permanent residence in the selected ethnic minority-concentrated areas of Yunnan Province for at least 5 years; (3) diagnosis by a medical institution of at least one eligible chronic non-communicable disease, including hypertension, type 2 diabetes, coronary heart disease, stroke, or chronic obstructive pulmonary disease; (4) disease history of at least 6 months and a stable condition at the time of survey, without acute exacerbation or hospitalization; (5) mental alertness and ability to understand and complete the questionnaire independently or with standardized investigator assistance. These disease groups were analyzed together because the study focused on general health-behavior associations among adults with chronic diseases rather than disease-specific clinical effects. Although these conditions differ clinically, they share long-term needs for lifestyle management, physical activity guidance, psychological adjustment, and chronic disease self-management.

Exclusion criteria were as follows: (1) severe cognitive impairment, severe psychiatric disorder, or other conditions affecting comprehension and valid response; (2) severe visual, hearing, or language impairment preventing questionnaire completion even with standardized assistance; (3) acute exacerbation of chronic disease, severe complications, or current hospitalization; (4) residence in the study area for less than 5 years; (5) questionnaires with substantial missing data, logical inconsistencies, or duplicate responses.

The sample size was estimated using the formula for a population proportion:

$$N=\frac{Z_{\alpha /2}^{2}P(1-P)}{\delta^{2}}$$

where P represents the expected proportion of the key study characteristic and δ represents the allowable error. Because no prior study had reported this proportion specifically among adults with chronic diseases in multiethnic areas of Yunnan Province, P was set at 0.558 based on a previous study in a comparable Yunnan setting (23), with α = 0.05, Z_α/2_ = 1.96, and δ = 0.03. The initial sample size was approximately 1,053. Considering the multistage stratified sampling framework, a design effect of 1.5 was applied. After allowing for a 15% invalid questionnaire rate, the minimum required sample size was calculated as

$$N=\frac{1053\times 1.5}{1-0.15}=1858$$

A total of 1,988 valid questionnaires were included in the final analysis, exceeding the minimum requirement.

A total of 1,988 participants were included, including 1,191 men (59.9%) and 797 women (40.1%). The ethnic composition was 322 Han (16.1%), 481 Tibetan (24.1%), 327 Yi (16.4%), 235 Bai (11.8%), 360 Lisu (18.1%), 219 Naxi (11.0%), and 44 participants from other ethnic groups (2.2%). Sociodemographic characteristics are shown in Table 1, and PA and HPL levels are shown in Table 2.

**Table 1:** Sociodemographic characteristics of the participants (*N* = 1,988).

Categorical variable	Category	N (%)
Gender	Male	1,191 (59.9)
	Female	797 (40.1)
Age group(years)	Young (18–44)	724 (36.4)
	middle-aged (45–59)	1,069 (53.8)
	older adult (≥60)	195 (9.8)
Educational	Below primary school	37 (1.9)
	Junior high school	386 (19.4)
	Senior high school	526 (26.5)
	Junior college	464 (23.3)
	Bachelor’s degree	501 (25.2)
	Postgraduate or above	74 (3.7)
Marital	Single	312 (15.9)
	Married	1,481 (74.5)
	Divorced	103 (5.2)
	Widowed	65 (3.3)
	Prefer not to disclose	27 (1.4)
Employment	Unemployed	400 (20.1)
	Employed	1,131 (56.9)
	Retired	457 (23.0)
Smoking and drinking history	Yes	1,388 (69.8)
	No	600 (30.2)
Ethnic group	Han	322 (16.1)
	Tibetan	481 (24.1)
	Yi	327 (16.4)
	Bai	235 (11.8)
	Lisu	360 (18.1)
	Naxi	219 (11.0)
	Other ethnicities	44 (2.2)

**Table 2:** Distribution of PA and HPL levels among participants (*N* = 1,988).

Variable	Category	N (%)
PARS-3	High level	162 (8.1)
	Moderate level	150 (7.5)
	Low level	1,676 (84.3)
HPLP-IIR	Low	788 (39.6)
	Pass	501 (25.2)
	Good	399 (20.1)
	Excellent	299 (15.0)

### 2.2 Measures

In addition to previous psychometric evidence for the Chinese versions of these instruments, supplementary psychometric analyses were conducted in the present sample, including internal consistency, KMO, Bartlett’s test of sphericity, and exploratory factor analysis. Because item-level HPLP-IIR responses were not retained in the final analytical dataset, structural evaluation of the HPLP-IIR was based on its six dimension scores. Given that the PARS-3 comprises three directly observed behavioral indicators, its factor-analytic results were interpreted as exploratory. Exploratory subgroup analyses across the six major ethnic groups were also conducted to assess instrument applicability in this multiethnic sample.

#### 2.2.1 Health-promoting lifestyle profile-II revised

The Health-promoting Lifestyle Profile-II Revised (HPLP-IIR) was used to assess health-promoting behaviors (2). The Chinese version has shown acceptable construct validity (24). The scale includes 52 items across six dimensions: interpersonal relations, health responsibility, stress management, nutrition, exercise, and spiritual growth. Each item is scored on a 4-point Likert scale from 1 (“never”) to 4 (“routinely”), with total scores ranging from 52 to 208. Higher scores indicate a healthier lifestyle, and scores are classified as low (52–90), pass (91–129), good (130–169), and excellent (170–208). In the present sample, structural evaluation was based on the six dimension scores because item-level HPLP-IIR responses were not retained. The six dimension scores showed excellent internal consistency (Cronbach’s *α* = 0.984), excellent sampling adequacy (KMO = 0.942), and a significant Bartlett’s test of sphericity (χ^2^ = 21391.72, df = 15, *p* < 0.001). Exploratory factor analysis extracted one dominant factor with an eigenvalue of 5.57, explaining 92.85% of the variance, with dimension loadings from 0.960 to 0.971. Across the six major ethnic groups, Cronbach’s α ranged from 0.954 to 0.991.

#### 2.2.2 Physical activity rating scale

The Physical Activity Rating Scale (PARS-3) was used to assess physical activity level (25). It includes three directly observed components: exercise intensity, duration, and frequency. The total score is calculated as intensity × (duration − 1) × frequency, with a possible range of 0–100. Physical activity is classified as low (≤19), moderate (20–42), or high (≥43). Because the PARS-3 comprises three behavioral indicators, it was treated as a composite index of physical activity. In the present sample, internal consistency was acceptable (Cronbach’s *α* = 0.761), KMO was 0.620, and Bartlett’s test was significant (χ^2^ = 1895.33, df = 3, *p* < 0.001). Exploratory factor analysis extracted one factor with an eigenvalue of 2.04, explaining 68.09% of the variance, with loadings from 0.697 to 0.897. These factor-analytic results should be interpreted as exploratory. Across the six major ethnic groups, Cronbach’s α ranged from 0.637 to 0.806.

#### 2.2.3 World health organization-5 well-being index

The World Health Organization-5 Well-Being Index (WHO-5) was used to assess subjective well-being (26). It includes five items covering cheerfulness, calmness, vitality, restfulness, and interest in daily life. Each item is scored from 0 (“at no time”) to 5 (“all of the time”), with a total score ranging from 0 to 25; higher scores indicate higher SWB, and scores ≤13 suggest possible depressive mood or lower quality of life. Previous validation of the Chinese version supported a unidimensional structure and good construct validity in patients with type 2 diabetes (26). In the present sample, internal consistency was excellent (Cronbach’s *α* = 0.979), KMO was 0.873, and Bartlett’s test was significant (χ^2^ = 16291.28, df = 10, *p* < 0.001). Exploratory factor analysis supported a one-factor structure, with an eigenvalue of 4.61 explaining 92.18% of the variance and item loadings from 0.956 to 0.964. Across the six major ethnic groups, Cronbach’s α ranged from 0.956 to 0.984.

#### 2.2.4 Self-efficacy for managing chronic disease 6-item scale

The Self-Efficacy for Managing Chronic Disease 6-Item Scale (SEMCD-6) was used to assess self-efficacy to manage chronic disease (27). The scale includes six items covering symptom coping and general disease management self-efficacy. Each item is scored from 1 (“not at all confident”) to 10 (“totally confident”), and the final score is calculated as the mean of all six items; higher scores indicate better SEMCD (28). In the present sample, internal consistency was excellent (Cronbach’s *α* = 0.996), KMO was 0.902, and Bartlett’s test was significant (χ^2^ = 36919.83, df = 15, *p* < 0.001). Exploratory factor analysis supported a strong general factor, with an eigenvalue of 5.90 explaining 98.28% of the variance and item loadings from 0.989 to 0.992. Across the six major ethnic groups, Cronbach’s α ranged from 0.996 to 0.997.

### 2.3 Definition and coding of variables

Sex was coded as male = 1 and female = 0. Age was coded as young (18–44 years) = 0, middle-aged (45–59 years) = 1, and older adult (≥60 years) = 2. Ethnicity was coded as Han = 1, Tibetan = 2, Yi = 3, Bai = 4, Lisu = 5, Naxi = 6, and other ethnicities = 7. Educational level was coded as primary school or below = 0, junior high school = 1, senior high school = 2, junior college = 3, bachelor’s degree = 4, and master’s degree or above = 5. Employment status was coded as unemployed = 0, employed = 1, and retired = 2. Marital status was coded as single = 0, married = 1, divorced = 2, widowed = 3, and prefer not to disclose = 4. Smoking and drinking history was coded as yes = 1 and no = 0, with “yes” defined as current smoking or drinking or such behavior within the past 12 months. PA, SWB, SEMCD, and HPL were operationalized using the PARS-3, WHO-5, SEMCD-6, and HPLP-IIR scores, respectively.

### 2.4 Statistical analysis

Data were analyzed using SPSS 26.0 and the PROCESS macro developed by Hayes (29). To reduce common method bias at the procedural level, standardized investigator training, uniform instructions, voluntary participation, confidentiality protection, de-identification, IP restriction, and independent questionnaire completion were implemented. Harman’s single-factor test (30) was used as an initial statistical diagnostic. Because all main variables were self-reported and collected at the same time, a supplementary full collinearity assessment was also conducted. Each main construct score, including PA, SWB, SEMCD, and HPL, was regressed on the other construct scores, and variance inflation factor (VIF) values below 3.3 were considered to indicate that serious common method bias was unlikely. These analyses were interpreted as diagnostic evidence only, and residual common method bias was acknowledged in the limitations.

As supplementary psychometric analyses, internal consistency, KMO, Bartlett’s test of sphericity, and exploratory factor analysis were conducted for the WHO-5, SEMCD-6, PARS-3, and the six HPLP-IIR dimension scores. Exploratory subgroup reliability analyses were also conducted across the six major ethnic groups. Formal measurement invariance testing was not conducted because the final dataset did not retain item-level HPLP-IIR responses, the PARS-3 was treated as a three-component behavioral composite rather than a latent psychometric scale, and ethnic subgroup sizes were unbalanced, especially for the “other ethnicities” group. Therefore, subgroup reliability analyses were interpreted only as preliminary evidence of instrument applicability, and the lack of formal measurement invariance testing was acknowledged as a limitation.

Measurement data were expressed as mean ± standard deviation (x̄ ± s). Group differences were tested using independent-samples t tests or one-way ANOVA, and Pearson correlation analysis was used for continuous variables. To address potential conceptual overlap between PA measured by the PARS-3 and the exercise dimension of the HPLP-IIR, a sensitivity analysis was conducted by recalculating the HPLP-IIR score after excluding the exercise dimension. The non-exercise HPLP-IIR score was then used as the outcome variable to repeat the correlation, multivariable regression, and serial indirect association analyses. Covariates, including ethnicity, sex, age, educational level, marital status, employment status, and smoking/drinking history, were selected *a priori* based on previous literature and their relevance to the study variables.

After adjustment for covariates, regression analysis was used to examine associations among variables. Multicollinearity diagnostics showed VIF values of 1.08–1.80, indicating no serious multicollinearity. PROCESS Model 6 was used to examine the hypothesized PA → SWB → SEMCD → HPL serial indirect association model, with 5,000 bootstrap samples and 95% CIs. An indirect association was considered statistically significant when the 95% CI did not include zero. The significance level was *α* = 0.05. Given the cross-sectional design, PROCESS Model 6 was used only to estimate statistical indirect associations and was not interpreted as evidence of causal mediation or temporal ordering.

### 2.5 Procedure

All procedures complied with the 1964 Declaration of Helsinki. Before enrollment, trained investigators provided standardized information on the study purpose, procedures, duration, risks and benefits, confidentiality, data use, contact information, and the right to withdraw without affecting medical care. Written informed consent was obtained before questionnaire completion. For participants with low literacy, reading difficulties, or language communication difficulties, trained investigators provided standardized explanations in Mandarin with locally understandable language support when needed, and participation proceeded only after comprehension and voluntary consent were confirmed. Participants were instructed to complete the questionnaire independently whenever possible. Individuals unable to provide valid responses because of severe cognitive, psychiatric, communication, or acute medical conditions were excluded. All data were de-identified and stored with ethics-required access control. Ethical approval was granted by the Ethics Committee of Southwest University Hospital (SWU-ETF-2023-07-17-011).

## 3 Results

### 3.1 Common method bias analysis

Harman’s single-factor test was used as an initial diagnostic for common method bias. Unrotated exploratory factor analysis showed that the first common factor explained 32.02% of the variance, below the empirical threshold of 40% (31). The supplementary full collinearity assessment showed VIF values of 1.065 for PA, 1.218 for SWB, 1.213 for SEMCD, and 1.238 for HPL, all below the recommended threshold of 3.3. These results did not indicate serious common method bias, although common method bias cannot be completely ruled out because all main variables were self-reported and collected at the same time.

### 3.2 Descriptive and comparative analyses

#### 3.2.1 Descriptive analysis

The mean total PARS-3 score was 10.22 ± 19.92, indicating generally low physical activity. The mean scores for exercise intensity, duration, and frequency were 1.59 ± 0.88, 2.31 ± 1.39, and 2.44 ± 1.42, respectively. The mean total HPLP-IIR score was 114.94 ± 39.28, indicating a passable level of HPL. Among the six HPL dimensions, interpersonal relations scored highest, followed by spiritual growth, nutrition, health responsibility, stress management, and exercise (Table 3).

**Table 3:** Descriptive statistics for PA and HPL.

Categorical variable	M ± SD	Dimension	M ± SD
PARS-3	10.22 ± 19.92	Exercise intensity Exercise duration Exercise frequency	1.59 ± 0.88 2.31 ± 1.39 2.44 ± 1.42
HPLP-IIR	114.94 ± 39.28	Spiritual growth Health responsibility Exercise Nutrition Interpersonal relations Stress management	20.40 ± 7.68 19.24 ± 6.67 16.46 ± 6.67 20.20 ± 6.28 20.80 ± 7.10 17.83 ± 6.33

#### 3.2.2 Sociodemographic differences in health-promoting lifestyle

HPL scores differed significantly by age, ethnicity, marital status, employment status, smoking/drinking history, and PA level (Table 4). Older adults scored higher than young and middle-aged adults (*F* = 97.083, *p* < 0.001). HPL also differed across ethnic groups (*F* = 17.210, *p* < 0.001), with the Han group scoring higher than the Tibetan, Yi, Bai, Lisu, and Naxi groups. Significant differences were also observed by marital status (*F* = 8.552, *p* < 0.001), employment status (*F* = 68.697, *p* < 0.001), and smoking/drinking history (*t* = 2.458, *p* = 0.014). Participants with high PA levels had higher HPL scores than those with low or moderate PA levels (*F* = 54.760, *p* < 0.001).

**Table 4:** Sociodemographic characteristics of health-promoting lifestyle.

Categorical variable	Category	HPL (*M ± SD*)	*t/F*	*p*
Gender	Male Female	114.43 ± 39.42 115.71 ± 39.07	0.709	0.479
Age group(years)	Young (18–44) middle-aged (45–59) older adult (≥60)	106.35 ± 41.91 114.64 ± 35.10 148.51 ± 32.69	97.083	<0.001
Ethnic group	Han Tibetan Yi Bai Lisu Naxi Other ethnicities	129.79 ± 27.81 113.23 ± 38.00 117.83 ± 41.95 105.91 ± 44.05 113.67 ± 38.48 100.83 ± 40.16 132.43 ± 38.06	17.210	<0.001
Educational	Below primary school Junior high school Senior high school Junior college Bachelor’s degree Postgraduate or above	119.78 ± 36.20 116.20 ± 37.18 116.90 ± 40.59 114.10 ± 40.21 113.17 ± 39.36 109.27 ± 35.30	1.011	0.410
Marital	Single Married Divorced Widowed Prefer not to disclose	103.70 ± 42.87 117.29 ± 38.11 110.96 ± 38.09 117.95 ± 40.21 123.81 ± 39.67	8.552	<0.001
Employment	Unemployed Employed Retired	109.00 ± 42.11 109.66 ± 37.63 133.23 ± 35.01	68.697	<0.001
Smoking and drinking history	Yes No	113.52 ± 39.81 118.23 ± 37.86	2.458	0.014
PARS-3	Low level Moderate level High level	113.30 ± 36.64 102.40 ± 37.23 143.51 ± 52.94	54.76	<0.001

### 3.3 Correlation analysis

Pearson correlation analysis showed significant positive correlations among HPL, PA, SEMCD, and SWB (all *p* < 0.01). Specifically, HPL was positively correlated with PA (*r* = 0.203), SEMCD (*r* = 0.337), and SWB (*r* = 0.352). PA was positively correlated with SEMCD (*r* = 0.189) and SWB (*r* = 0.158), and SEMCD was positively correlated with SWB (*r* = 0.332). These results supported subsequent regression and serial indirect association analyses (Table 5).

**Table 5:** Correlation analysis of HPL, PA, SEMCD, and SWB.

Variable	HPL	PA	SEMCD	SWB
HPL	-			
PA	0.203^**^	-		
SEMCD	0.337^**^	0.189^**^	-	
SWB	0.352^**^	0.158^**^	0.332^**^	-

Values are Pearson correlation coefficients. ^**^p < 0.01.

### 3.4 Regression analysis

After adjustment for ethnicity, gender, age, educational level, marital status, employment status, and smoking/drinking history, PA was positively associated with HPL in Model 1 (standardized *β* = 0.286, *t* = 13.422, *p* < 0.001) and with SWB in Model 2 (standardized *β* = 0.207, *t* = 9.232, *p* < 0.001). In Model 3, PA (standardized *β* = 0.141, *t* = 6.393, *p* < 0.001) and SWB (standardized *β* = 0.308, *t* = 14.222, *p* < 0.001) were positively associated with SEMCD. In Model 4, PA (standardized *β* = 0.198, *t* = 9.661, *p* < 0.001), SWB (standardized *β* = 0.201, *t* = 9.604, *p* < 0.001), and SEMCD (standardized *β* = 0.229, *t* = 11.092, *p* < 0.001) were positively associated with HPL. Detailed standardized coefficients with 95% CIs are shown in Table 6.

**Table 6:** Regression models for HPL, SWB, and SEMCD.

Model	Outcome variable	Predictor variable	Standardized β (95% CI)	t	*p*	R^2^	F
Model 1	HPL	PA	0.286 (0.245, 0.328)	13.422	<0.001	0.156	45.665
		Ethnic group	−0.078 (−0.120, −0.036)	−3.651	<0.001		
		Gender	0.009 (−0.043, 0.060)	0.329	0.742		
		Age group	0.234 (0.182, 0.287)	8.806	<0.001		
		Educational	−0.030 (−0.073, 0.013)	−1.360	0.174		
		Marital	0.012 (−0.031, 0.056)	0.553	0.580		
		Employment	0.114 (0.062, 0.167)	4.272	<0.001		
		Smoking and drinking history	0.001 (−0.053, 0.056)	0.045	0.964		
Model 2	SWB	PA	0.207 (0.163, 0.251)	9.232	<0.001	0.066	17.476
		Ethnic group	−0.058 (−0.102, −0.014)	−2.568	0.010		
		Gender	0.009 (−0.045, 0.063)	0.320	0.749		
		Age group	0.112 (0.057, 0.167)	4.002	<0.001		
		Educational	−0.024 (−0.069, 0.022)	−1.023	0.307		
		Marital	0.033 (−0.013, 0.079)	1.420	0.156		
		Employment	0.071 (0.016, 0.126)	2.519	0.012		
		Smoking and drinking history	−0.032 (−0.090, 0.025)	−1.113	0.266		
Model 3	SEMCD	PA	0.141 (0.098, 0.185)	6.393	<0.001	0.132	33.407
		SWB	0.308 (0.266, 0.351)	14.222	<0.001		
		Ethnic group	0.020 (−0.023, 0.062)	0.909	0.364		
		Gender	0.001 (−0.051, 0.053)	0.042	0.967		
		Age group	−0.032 (−0.085, 0.022)	−1.163	0.245		
		Educational	−0.006 (−0.050, 0.038)	−0.249	0.803		
		Marital	0.001 (−0.043, 0.045)	0.049	0.961		
		Employment	0.050 (−0.004, 0.103)	1.828	0.068		
		Smoking and drinking history	−0.019 (−0.074, 0.036)	−0.681	0.496		
Model 4	HPL	PA	0.198 (0.158, 0.238)	9.661	<0.001	0.270	73.080
		SWB	0.201 (0.160, 0.241)	9.604	<0.001		
		SEMCD	0.229 (0.188, 0.269)	11.092	<0.001		
		Ethnic group	−0.067 (−0.106, −0.028)	−3.358	<0.001		
		Gender	0.006 (−0.042, 0.054)	0.245	0.807		
		Age group	0.211 (0.162, 0.260)	8.492	<0.001		
		Educational	−0.022 (−0.063, 0.018)	−1.085	0.278		
		Marital	0.003 (−0.037, 0.043)	0.147	0.883		
		Employment	0.084 (0.035, 0.133)	3.354	<0.001		
		Smoking and drinking history	0.014 (−0.036, 0.065)	0.559	0.576		

PA, SWB, SEMCD, and HPL were operationalized using the PARS-3, WHO-5, SEMCD-6, and HPLP-IIR scores, respectively. Standardized β values with 95% CIs are reported. Model 1 examined the association of PA with HPL after adjustment for covariates. Model 2 examined the association of PA with SWB. Model 3 examined the associations of PA and SWB with SEMCD. Model 4 examined the associations of PA, SWB, and SEMCD with HPL after adjustment for covariates. All models were adjusted for ethnicity, sex, age group, educational level, marital status, employment status, and smoking/drinking history where applicable. *p* values are reported as exact values when *p* ≥ 0.001 and as <0.001 when smaller.

**Figure 3:**
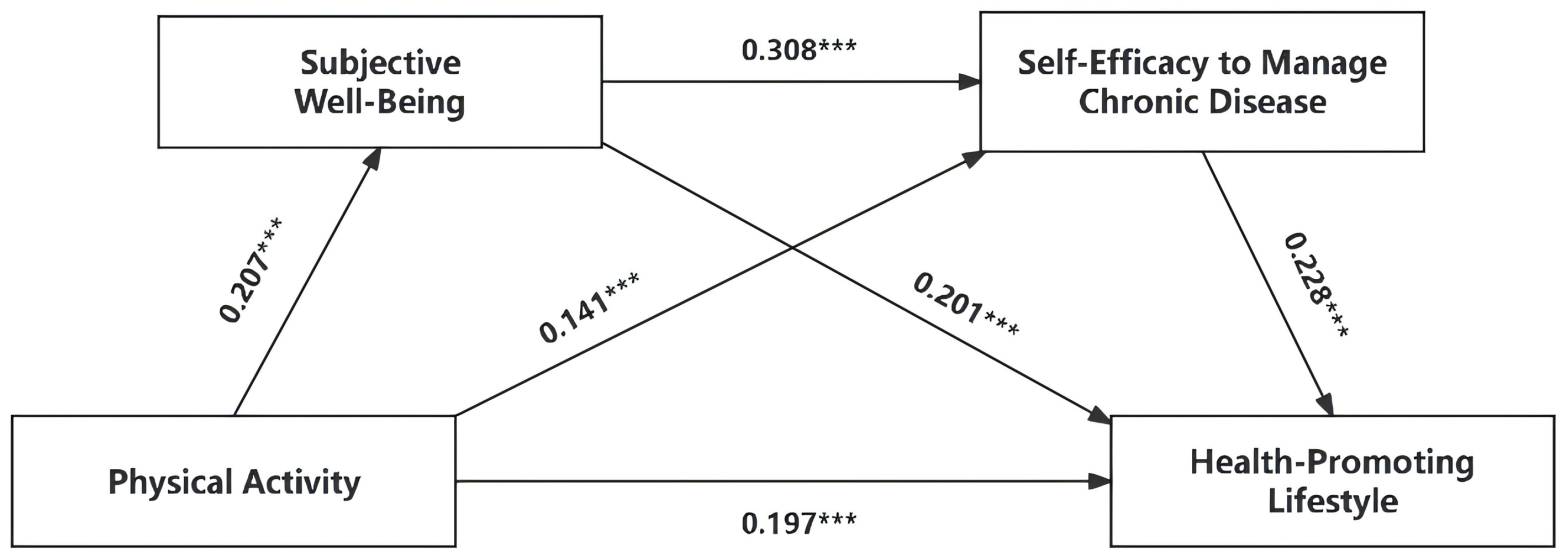
Serial indirect association model diagram.

The serial indirect association analysis showed a total association of PA with HPL of 0.565 [Boot SE = 0.042, 95% CI: 0.482, 0.647]. The total indirect association involving SWB and SEMCD was 0.174 [Boot SE = 0.014, 95% CI: 0.147, 0.202], accounting for 30.90% of the total association. The indirect association through SWB was 0.082 [Boot SE = 0.009, 95% CI: 0.064, 0.101], the indirect association through SEMCD was 0.064 [Boot SE = 0.009, 95% CI: 0.046, 0.083], and the serial indirect association through PA → SWB → SEMCD → HPL was 0.029 [Boot SE = 0.004, 95% CI: 0.022, 0.037]. All 95% CIs excluded zero, supporting significant indirect associations within the prespecified cross-sectional statistical model (Table 7; Figure 3).

**Table 7:** Indirect associations in the serial indirect association model.

Pathway	Estimate	Boot SE (95% CI)	Proportion of total association (%)
PA → SWB → HPL	0.082	0.009 (0.064, 0.101)	14.53
PA → SEMCD → HPL	0.064	0.009 (0.046, 0.083)	11.34
PA → SWB → SEMCD → HPL	0.029	0.004 (0.022, 0.037)	5.14
Total indirect association	0.174	0.014 (0.147, 0.202)	30.90
Total association	0.565	0.042 (0.482, 0.647)	100.00

Estimates are unstandardized bootstrap estimates. Boot SE, bootstrap standard error; CI, confidence interval. The model was adjusted for ethnicity, sex, age group, educational level, marital status, employment status, and smoking/drinking history.

To address the potential conceptual overlap between PARS-3 and the HPLP-IIR exercise dimension, the analyses were repeated using the non-exercise HPLP-IIR score as the outcome. After excluding the exercise dimension, PA remained correlated with the non-exercise HPLP-IIR score (*r* = 0.201, *p* < 0.001). In the adjusted regression model, PA (*β* = 0.196, *t* = 9.578, *p* < 0.001), SWB (*β* = 0.204, *t* = 9.775, *p* < 0.001), and SEMCD (*β* = 0.228, *t* = 11.073, *p* < 0.001) remained positively associated with the non-exercise HPLP-IIR score. The serial indirect association analysis also remained significant, with a total association of 0.471 and a total indirect association of 0.147, accounting for 31.24% of the total association. These findings suggest that the main results were not solely driven by shared exercise-related content. Detailed results are provided in the Supplementary material.

## 4 Discussion

This study examined the association between PA and HPL and the statistical involvement of SWB and SEMCD among adults with chronic diseases in multiethnic areas of Yunnan Province, China. PA and HPL were both relatively low, and HPL varied across several sociodemographic subgroups. The PA–HPL association was accompanied by significant indirect associations involving SWB and SEMCD. The discussion focuses on possible contextual explanations and the theoretical implications of this serial indirect association pattern.

PA and HPL were relatively low in this sample, with weaker performance in exercise and stress management. This may reflect constrained opportunities for sustained exercise and structured health management in multiethnic areas of Yunnan because of complex terrain, uneven accessibility of health services, and insufficient exercise guidance (11), as well as limited chronic disease health literacy (10, 12). This interpretation is consistent with evidence showing high prevalence of insufficient PA among Chinese adults (32) and poor exercise adherence among patients with chronic diseases (33, 34). The relatively higher scores for interpersonal relations and spiritual growth may be partly related to community-level social interaction and collective cultural activities reported in some multiethnic settings (35, 36). However, this explanation remains exploratory because social network structure and cultural participation were not directly measured.

HPL also varied across age, marital status, employment status, and smoking/drinking history. Older and retired participants had higher HPL scores. Although this pattern differs from some previous findings in China (37), it may be related to more stable daily routines and greater discretionary time for exercise, dietary regulation, medication management, and other health-promoting behaviors (38, 39). In some ethnic minority communities in Yunnan, older adults may also have greater access to family, neighborhood, and community support, which could facilitate daily health management (40). Recent research from ethnic minority groups in Yunnan further indicates that social capital and social support are associated with healthier eating patterns and health-related knowledge (41). Young and middle-aged adults may face competing demands from employment, household responsibilities, and migration-related livelihood pressures, which may reduce the consistency of health-promoting behaviors (42). Married participants had higher HPL scores than unmarried participants, consistent with evidence that marriage may support healthy lifestyles through dietary care, medical visit accompaniment, emotional support, and behavioral monitoring (43). Lower HPL among unemployed participants may be related to economic pressure and lack of social roles (44). Participants without smoking or drinking history also had higher HPL scores, which is consistent with evidence on the combined health effects of lifestyle behaviors (45). In terms of ethnic differences, the Han group showed higher HPL scores overall, whereas lower scores were observed among Tibetan, Yi, Bai, Lisu, and Naxi participants. These differences should be interpreted cautiously and should not be attributed to ethnicity itself. Previous studies suggest that health behaviors in multiethnic areas may be shaped by geographic location, economic conditions, health service access, dietary patterns, cultural practices, and disease perception (46). Therefore, the observed ethnic differences may reflect broader contextual and social determinants rather than inherent characteristics of any ethnic group. Regional studies in Yunnan have described high-salt dietary practices among Bai residents (47), high-fat traditional dietary patterns among Tibetan residents living in high-altitude environments (48), and tobacco exposure in some rural ethnic minority areas (49). Because unhealthy dietary patterns are associated with chronic disease risk, these regional dietary characteristics may partly explain differences in HPL patterns (50). Other studies have reported limited health awareness or self-rated health challenges among Yi and Lisu populations in specific rural settings (51). These findings provide contextual explanations but should not be generalized to all members of these ethnic groups. Future research should measure cultural, environmental, and socioeconomic factors more precisely rather than relying only on broad ethnic categories.

PA, SWB, and SEMCD were positively correlated with one another and were each positively associated with HPL in the regression models. Regular PA has been associated with lower depression and anxiety and higher positive affect and life satisfaction, potentially through better cardiopulmonary function, metabolic status, and sleep quality (17, 52). In this study, higher PA was linked to higher SWB among adults with chronic diseases in multiethnic areas (53). PA and SWB were also positively associated with SEMCD. This pattern is consistent with evidence that regular PA may be related to stronger confidence in chronic disease self-management (16), while higher SWB may be linked to more optimistic expectations and stronger perceived behavioral control (20). SEMCD has been associated with medication adherence, dietary modification, blood pressure and blood glucose monitoring, and exercise implementation (54), and its positive association with HPL was consistent with previous findings (21).

From the perspective of Social Cognitive Theory, the observed serial indirect association pattern is theoretically plausible. Regular PA may be accompanied by better bodily function, symptom relief, and positive affective feedback, which may be associated with higher SWB. Higher SWB may be related to stronger positive expectations, perceived control, and willingness to invest effort in chronic disease management, which may be associated with higher SEMCD. In turn, higher SEMCD may be linked to greater adherence to exercise, dietary regulation, symptom monitoring, and other health-promoting behaviors. Thus, SWB may represent an affective correlate, whereas SEMCD may represent a more proximal cognitive-behavioral correlate in the PA–HPL association.

The indirect association pattern suggests that the relationship between PA and HPL may involve multiple interconnected psychological correlates. The PA → SWB → HPL pathway indicates that higher PA was associated with higher SWB, which was further associated with higher HPL. The PA → SEMCD→HPL pathway indicates that higher PA was associated with stronger SEMCD, which was further associated with higher HPL. The serial PA → SWB → SEMCD→HPL pathway indicates a sequential statistical association pattern. Regular PA has been associated with stronger self-efficacy for health-related behavior maintenance (13). Higher SWB may also be linked to stronger perceived control and greater confidence in chronic disease self-management (16). Better SEMCD may then be associated with healthier daily behavior patterns and stronger HPL maintenance (55). These findings provide an association-based explanation for the observed PA–HPL relationship and require confirmation in longitudinal or intervention studies. From a practical perspective, intervention design for multiethnic areas should move beyond general health promotion and adopt culturally adapted strategies.

Practically, PA promotion may be embedded in culturally familiar group-based activities, such as ethnic traditional dance, sport forms (56), or adapted traditional Chinese exercises (57), to support participation and adherence. Chronic disease health education should be delivered in Mandarin together with locally used ethnic languages or dialect-supported audiovisual materials, especially for older adults and individuals with limited health literacy (58, 59). Because family and community support remain important in Yunnan’s ethnic minority settings (40, 41), village doctors, community health workers, family members, and local peers may be incorporated into follow-up, symptom monitoring, and behavioral support.

## 5 Limitations

Several limitations should be acknowledged. First, this cross-sectional study cannot establish the temporal ordering of PA, SWB, SEMCD, and HPL, and the results should not be interpreted as causal mediation, mechanisms, or temporal pathways. Longitudinal and intervention studies are needed to verify the observed association pattern. Second, all main variables were self-reported and collected at a single time point, which may introduce recall bias, social desirability bias, reporting inaccuracy, and potential common method bias. Although Harman’s single-factor test and the supplementary full collinearity assessment did not suggest serious common method bias, these diagnostics cannot definitively exclude this concern. Future studies should incorporate objective PA measures, fitness testing, and clinical or medical record verification. Third, the HPLP-IIR includes an exercise dimension that is conceptually adjacent to PA measured by the PARS-3. Sensitivity analyses using the non-exercise HPLP-IIR score showed stable results, suggesting that the PA–HPL association was not solely attributable to shared exercise-related content. Fourth, the sampling procedure used a multistage stratified site-based framework with quota-based participant recruitment. Randomization was mainly applied at the geographic and site-selection levels, while individual recruitment depended on eligibility screening, voluntary participation, and quota completion; therefore, selection bias may have occurred, and generalization should be cautious. Fifth, different chronic diseases were analyzed together. Although hypertension, type 2 diabetes, coronary heart disease, stroke, and chronic obstructive pulmonary disease share long-term needs for lifestyle management and self-management support, they may differ in physical activity capacity, symptom burden, treatment restrictions, self-efficacy, and well-being. Future studies should conduct disease-specific subgroup or stratified analyses. Sixth, ethnicity was treated as a background variable, but deeper cultural factors, including health beliefs, lifestyle norms, dietary practices, language use, social support, and culturally specific understandings of physical activity and self-management, were not directly measured. Therefore, cultural explanations should be interpreted cautiously as contextual interpretations rather than direct evidence from this dataset. Finally, formal measurement invariance testing across ethnic groups was not conducted. Although supplementary psychometric analyses supported preliminary instrument applicability, item-level HPLP-IIR responses were not retained, PARS-3 was treated as a behavioral composite, and ethnic subgroup sizes were unbalanced. Future studies should retain item-level responses and conduct multi-group confirmatory factor analysis to test measurement invariance across ethnic groups.

## 6 Conclusion

In summary, adults with chronic diseases in multiethnic areas of Yunnan Province showed low PA and moderately low HPL, particularly in the exercise and stress management dimensions. HPL differed by age, ethnicity, marital status, employment status, and smoking/alcohol history. PA, SWB, and SEMCD were positively correlated with one another and were each positively associated with HPL. The PA–HPL association was accompanied by significant indirect associations involving SWB and SEMCD, supporting the hypothesized PA–SWB–SEMCD–HPL association pattern. These findings suggest that health-promotion programs for chronic disease populations in multiethnic regions may consider culturally adapted PA promotion, language-appropriate chronic disease education, and community-supported self-management. Because this study was cross-sectional, these implications should be considered hypothesis-generating and require confirmation in longitudinal or intervention research.

## Data Availability

The datasets used and/or analyzed in the current study are not publicly available because they contain human participant data and are subject to ethical and privacy restrictions. De-identified data may be made available from the corresponding author upon reasonable request, subject to approval by the Ethics Committee of Southwest University Hospital and in accordance with participant confidentiality requirements. Requests to access the datasets should be directed to Li Peng, 804455169@qq.com.

## Footnotes

1
https://www.wjx.cn/

## References

[1] Wang L Peng W Zhao Z Zhang M Shi Z Song Z . Prevalence and treatment of diabetes in China, 2013-2018. JAMA. (2021) 326:2498–506. doi: 10.1001/jama.2021.22208, 34962526 PMC8715349

[2] Cao WJ Guo Y Ping WW Zheng JZ. Development and psychometric testing of the Chinese version of the HPLP-II. Chin J Dis Control Prev. (2016) 20:286–9. doi: 10.16462/j.cnki.zhjbkz.2016.03.018

[3] Li Y Pan A Wang DD Liu X Dhana K Franco OH . Impact of healthy lifestyle factors on life expectancies in the US population. Circulation. (2018) 138:345–55. doi: 10.1161/CIRCULATIONAHA.117.032047, 29712712 PMC6207481

[4] Diener E Oishi S Tay L. Advances in subjective well-being research. Nat Hum Behav. (2018) 2:253–60. doi: 10.1038/s41562-018-0307-6, 30936533

[5] Piercy KL Troiano RP Ballard RM Carlson SA Fulton JE Galuska DA . The physical activity guidelines for Americans. JAMA. (2018) 320:2020–8. doi: 10.1001/jama.2018.14854, 30418471 PMC9582631

[6] Warburton DER Bredin SSD. Health benefits of physical activity: a systematic review of current systematic reviews. Curr Opin Cardiol. (2017) 32:541–56. doi: 10.1097/HCO.0000000000000437, 28708630

[7] Zhang Z Chen W. A systematic review of the relationship between physical activity and happiness. J Happiness Stud. (2019) 20:1305–22. doi: 10.1007/s10902-018-9976-0

[8] Ouyang Y Wang H He Y Su C Zhang J Du W . Physical activity and sedentary behavior among Chinese adults—10 PLADs, China, 2022–2023. China CDC Wkly. (2025) 7:6–9. doi: 10.46234/ccdcw2025.002, 39801822 PMC11718375

[9] Loprinzi PD Smit E Mahoney S. Physical activity and dietary behavior in US adults and their combined influence on health. Mayo Clin Proc. (2014) 89:190–8. doi: 10.1016/j.mayocp.2013.09.018, 24485132

[10] Zhao J Min XD Tang QL Zhang Q Wan R Liu ZT. Analysis of dietary knowledge, dietary attitudes, and dietary behaviors among residents in ethnic minority areas of Yunnan. Chin J Health Educ. (2023) 39:18–23,29. doi: 10.16168/j.cnki.issn.1002-9982.2023.01.004

[11] Chen J Wang XP Li XJ Zhang YF Huang Y Yan CF . Latent class analysis of health-promoting lifestyles among rural residents in ethnic minority-concentrated areas of Yunnan Province. Chin J Prev Med. (2024) 25:1259–64. doi: 10.16506/j.1009-6639.2024.10.006

[12] Hu HQ. Knowledge, Attitude, and Practice Pattern of Chronic Disease Residents in Lahu-Inhabited Areas of Yunnan and its Association With Health Status [Master’s Thesis]. Wuhan: Huazhong University of Science and Technology (2023).

[13] Bandura A. Self-Efficacy: The Exercise of Control. New York, NY: W.H. Freeman (1997).

[14] Lorig KR Sobel DS Ritter PL Laurent D Hobbs M. Effect of a self-management program on patients with chronic disease. Eff Clin Pract. (2001) 4:256–62. 11769298

[15] Du S Yuan C Xiao X Chu J Qiu Y Qian H. Self-management programs for chronic musculoskeletal pain conditions: a systematic review and meta-analysis. Patient Educ Couns. (2011) 85:e299–310. doi: 10.1016/j.pec.2011.02.021, 21458196

[16] McAuley E Blissmer B. Self-efficacy determinants and consequences of physical activity. Exerc Sport Sci Rev. (2000) 15:341–55. 10902091

[17] Schuch FB Vancampfort D Firth J Rosenbaum S Ward PB Silva ES . Physical activity and incident depression: a Meta-analysis of prospective cohort studies. Am J Psychiatry. (2018) 175:631–48. doi: 10.1176/appi.ajp.2018.17111194, 29690792

[18] Steptoe A Deaton A Stone AA. Subjective wellbeing, health, and ageing. Lancet. (2015) 385:640–8. doi: 10.1016/S0140-6736(13)61489-0, 25468152 PMC4339610

[19] Walker SN Sechrist KR Pender NJ. The health-promoting lifestyle profile: development and psychometric characteristics. Nurs Res. (1987) 36:76–81. 3644262

[20] Luo Z Zhong S Zheng S Li Y Guan Y Xu W . Influence of social support on subjective well-being of patients with chronic diseases in China: chain-mediating effect of self-efficacy and perceived stress. Front Public Health. (2023) 11:1184711. doi: 10.3389/fpubh.2023.1184711, 37427286 PMC10325675

[21] Amiri M Chaman R Khosravi A. The relationship between health-promoting lifestyle and its related factors with self-efficacy and well-being of students. Osong Public Health Res Perspect. (2019) 10:221–7. doi: 10.24171/j.phrp.2019.10.4.04, 31497493 PMC6711714

[22] Lu HP Li Q Hao HY Ma M Xue F Feng AF . Health behaviors and influencing factors among Mongolian adults with prehypertension. J Youjiang Med Univ Nationalities. (2021) 43:247–51.

[23] Cheng HZ. Current Status of Health-Promoting Lifestyle and Influencing Factors Among Rural Residents in Dehong Prefecture [master’s thesis.Dali University (2024).

[24] Teng H-L Yen M Fetzer S. Health promotion lifestyle profile-II: Chinese version short form. J Adv Nurs. (2010) 66:1864–73. doi: 10.1111/j.1365-2648.2010.05353.x, 20557380

[25] Liang DQ. The stress level of college students and its relationship with physical exercise. Chin Ment Health J. (1994) 8:5–6.

[26] Du J Jiang Y Lloyd C Sartorius N Ren J Zhao W . Validation of Chinese version of the 5-item WHO well-being index in type 2 diabetes mellitus patients. BMC Psychiatry. (2023) 23:890. doi: 10.1186/s12888-023-05381-9, 38031007 PMC10685601

[27] Ritter PL Lorig K. The English and Spanish self-efficacy to manage chronic disease scale measures were validated using multiple studies. J Clin Epidemiol. (2014) 67:1265–73. doi: 10.1016/j.jclinepi.2014.06.009, 25091546

[28] Chow SKY Wong FK. The reliability and validity of the Chinese version of the short-form chronic disease self-efficacy scales for older adults. J Clin Nurs. (2014) 23:1095–104. doi: 10.1111/jocn.12298, 23815418

[29] Hayes AF. Introduction to Mediation, Moderation, and Conditional Process Analysis: A Regression-Based Approach. 3rd ed. New York, NY: Guilford Press (2022).

[30] Kock N. Harman’s single factor test in PLS-SEM: checking for common method bias. Data Anal Perspect J. (2020) 2:1–6. doi: 10.1007/978-3-319-64069-3_11, 28118817 PMC5259953

[31] Chin WW Thatcher JB Wright RT. Assessing common method Bias: problems with the ULMC technique. MIS Q. (2012) 36:1003–19. doi: 10.2307/41703491

[32] Gao XX Wang LM Zhang X Zhao ZP Li C Huang ZJ . Prevalence and influencing factors of insufficient physical activity among Chinese adults in 2018. Chin J Epidemiol. (2023) 44:1190–7. doi: 10.3760/cma.j.cn112338-20221125-01000 37661608

[33] Wong MYC Ou KL Chung PK Chui KYK Zhang CQ. The relationship between physical activity, physical health, and mental health among older Chinese adults: a scoping review. Front Public Health. (2023) 10:914548. doi: 10.3389/fpubh.2022.914548, 36684983 PMC9853435

[34] Chen Y Zhu GY. Influencing factors and promotion strategies of physical activity in patients with chronic diseases: based on the capability, opportunity, motivation-behavior model. China Sport Sci Technol. (2024) 60:31–40. doi: 10.16470/j.csst.2024081

[35] Páez D Rimé B Basabe N Wlodarczyk A Zumeta L. Psychosocial effects of perceived emotional synchrony in collective gatherings. J Pers Soc Psychol. (2015) 108:711–29. doi: 10.1037/pspi0000014, 25822033

[36] Özgenel M Yılmaz Ö. The relationship between spiritual well-being and happiness: an investigation on teachers. Spirit Psychol Counsel. (2020) 5:287–300. doi: 10.37898/spc.2020.5.3.110

[37] Wang JJ Zhang MY Zhou XX Xie YY Zhao P Guo YY . Regional differences in health behaviors among patients with stroke and their influencing factors. Nurs Res. (2025) 39:3950–9. doi: 10.12102/j.issn.1009-6493.2025.23.007

[38] Potočnik K Sonnentag S. A longitudinal study of well-being in older workers and retirees: the role of engaging in different types of activities. J Occup Organ Psychol. (2013) 86:497–521. doi: 10.1111/joop.12003

[39] Hutchinson S Kleiber D. On time, leisure, and health in retirement: implications for public health services. Int J Environ Res Public Health. (2023) 20:2490. doi: 10.3390/ijerph20032490, 36767856 PMC9916207

[40] Zhang K Zakus D Gao C. Long-term care for aged ethnic minority people in Yunnan, China: understanding the situation. Fam Med Community Health. (2016) 4:64–8. doi: 10.15212/FMCH.2016.0119

[41] Zhang Q Huangfu C Wan Q Su W Zhu X Yu B . Social capital and healthy eating among two ethnic minority groups in Yunnan Province, Southwest China: the mediating role of social support and nutrition knowledge. Front Nutr. (2024) 11:1273851. doi: 10.3389/fnut.2024.1273851, 38883859 PMC11176612

[42] Kwasnicka D Dombrowski SU White M Sniehotta F. Theoretical explanations for maintenance of behaviour change: a systematic review of behaviour theories. Health Psychol Rev. (2016) 10:277–96. doi: 10.1080/17437199.2016.1151372, 26854092 PMC4975085

[43] Umberson D Karas MJ. Social relationships and health: a flashpoint for health policy. J Health Soc Behav. (2010) 51:S54–66. doi: 10.1177/0022146510383501, 20943583 PMC3150158

[44] Ullah P Banks M Warr P. Social support, social pressures and psychological distress during unemployment. Psychol Med. (1985) 15:283–95. 4023133 10.1017/s0033291700023564

[45] Loef M Walach H. The combined effects of healthy lifestyle behaviors on all cause mortality: a systematic review and meta-analysis. Prev Med. (2012) 55:163–70. doi: 10.1016/j.ypmed.2012.06.017, 22735042

[46] Lan Y Yang JY Shama Siji Jiang Q Qin JT Wu Q . Correlation between health-promoting lifestyle and depressive tendency among rural Yi older adults. Soft Sci Health. (2025) 39:45–50,61. doi: 10.3969/j.issn.1003-2800.2025.10.008

[47] Zhong YL. Bai dietary culture: equal emphasis on etiquette and delicacy. Ethnic Forum. (2003) 6:8. doi: 10.19683/j.cnki.mzlt.2003.06.006

[48] Li R Yuan L Xiong ZZ Xu XM Gou SY. Current status of glycemic control and influencing factors among Tibetan patients with type 2 diabetes. Nurs Res. (2016) 30:3609–12. doi: 10.3969/j.issn.1009-6493.2016.29.009

[49] Li GH Wang XM Li JB Liu L Mo Y Li Q . Smoking behaviors in three rural ethnic groups in Yunnan Province and their effects on chronic obstructive pulmonary disease. Modern Preventive Med. (2023) 50:2633–8. doi: 10.20043/j.cnki.MPM.202209590

[50] Jayedi A Soltani S Abdolshahi A Shab-Bidar S. Healthy and unhealthy dietary patterns and the risk of chronic disease: an umbrella review of meta-analyses of prospective cohort studies. Br J Nutr. (2020) 124:1133–44. doi: 10.1017/S0007114520002330, 32600500

[51] Hu QC Jiang LC Feng FJ Zhang YJ Zhao XM Zhang YY . Influencing factors of self-rated health among rural Yi older adults with hypertension in Yunnan. J Kunming Med Univ. (2023) 44:40–6. doi: 10.12259/j.issn.2095-610X.S20230704

[52] White RL Babic MJ Parker PD Lubans DR Astell-Burt T Lonsdale C. Domain-specific physical activity and mental health: a Meta-analysis. Am J Prev Med. (2017) 52:653–66. doi: 10.1016/j.amepre.2016.12.008, 28153647

[53] Marquez DX Aguiñaga S Vásquez PM Conroy DE Erickson KI Hillman C . A systematic review of physical activity and quality of life and well-being. Transl Behav Med. (2020) 10:1098–109. doi: 10.1093/tbm/ibz198, 33044541 PMC7752999

[54] Wang LT Wang Q Ma XL Song KY Wang J Wang GW . Relationship between health literacy and self-management behaviors in older adults with chronic multimorbidity: chain mediating effects of family health and self-efficacy. Modern Preventive Med. (2025) 52:3860–3865, 3892. doi: 10.20043/j.cnki.MPM.202505140

[55] Zhang LL Dong JQ. Research progress in self-management among patients with chronic diseases. Chinese J Prevention Control of Chronic Dis. (2010) 18:207–11. doi: 10.16386/j.cjpccd.issn.1004-6194.2010.02.005

[56] Joo JY Liu MF. Effectiveness of culturally tailored interventions for chronic illnesses among ethnic minorities. West J Nurs Res. (2021) 43:73–84. doi: 10.1177/0193945920918334, 32400300

[57] Zou L Xiao T Cao C Smith L Imm K Grabovac I . Tai chi for chronic illness management: synthesizing current evidence from meta-analyses of randomized controlled trials. Am J Med. (2021) 134:194–205.e12. doi: 10.1016/j.amjmed.2020.08.015, 32946848

[58] Hsueh L Hirsh AT Maupomé G Stewart JC. Patient–provider language concordance and health outcomes: a systematic review, evidence map, and research agenda. Med Care Res Rev. (2021) 78:3–23. doi: 10.1177/1077558719860708, 31291823

[59] Lor M Martinez GA. Scoping review: definitions and outcomes of patient-provider language concordance in healthcare. Patient Educ Couns. (2020) 103:1883–901. doi: 10.1016/j.pec.2020.05.025, 32507590

