# Supplementary material for: Physical activity and health-promoting lifestyles in adults with chronic diseases: serial indirect associations through subjective well-being and self-efficacy in multiethnic areas of Yunnan, China
Source: Front Public Health. 2026 May 29;14:1799074. doi: 10.3389/fpubh.2026.1799074 (PMC13260152; doi:10.3389/fpubh.2026.1799074)
Supplement: Supplementary file 1 [file Table_1.docx]

**Supplementary Material**

Supplementary sensitivity analysis addressing the potential conceptual proximity between PARS-3 and the HPLP-IIR total score

Supplementary sensitivity analysis result:

To address the concern regarding potential conceptual proximity between PARS-3 and the exercise-related dimension of the HPLP-IIR, we conducted a supplementary sensitivity analysis in which the six HPLP-IIR dimensions were analyzed separately as outcomes while keeping the original analytical framework unchanged. Pearson correlation analyses showed that PARS-3 was positively correlated with all six HPLP-IIR dimensions, including spiritual growth, health responsibility, physical activity, nutrition, interpersonal relations, and stress management, with all correlations reaching statistical significance (all P < 0.001). In multivariable linear regression models adjusted for ethnicity, sex, age group, educational level, marital status, employment status, and smoking/drinking history, PARS-3 remained significantly and positively associated with all six dimensions. The standardized regression coefficients ranged from 0.256 to 0.297, and all P values were below 0.001. Importantly, the associations of PARS-3 with health responsibility (β = 0.297) and nutrition (β = 0.295) were slightly stronger than that with the physical activity dimension itself (β = 0.279). These findings suggest that the association between PARS-3 and the overall HPLP-IIR score was not solely attributable to the shared exercise-related content of the two measures.

This supplementary analysis was designed to examine whether the observed association between PARS-3 and the total HPLP-IIR score was driven mainly by the exercise-related dimension embedded in the HPLP-IIR. The results showed that PARS-3 was significantly associated not only with the physical activity dimension, but also with all five non-exercise dimensions of the HPLP-IIR after adjustment for the same covariates used in the main model. Moreover, the strength of association for some non-exercise dimensions, particularly health responsibility and nutrition, was comparable to or slightly greater than that for the physical activity dimension. This pattern provides additional support that the main finding reflects a broader relationship between physical activity and multidimensional health-promoting lifestyle, rather than a purely mechanical overlap between conceptually adjacent exercise items.

Supplementary Table S1. Pearson correlations between PARS-3 and the six HPLP-IIR dimensions (N = 1,988)

| **Outcome dimension** | **r** | **P** |
| --- | --- | --- |
| Spiritual growth | 0.181 | <0.001 |
| Health responsibility | 0.220 | <0.001 |
| Physical activity | 0.201 | <0.001 |
| Nutrition | 0.227 | <0.001 |
| Interpersonal relations | 0.172 | <0.001 |
| Stress management | 0.187 | <0.001 |

Supplementary Table S2. Sensitivity analysis using the six HPLP-IIR dimensions as separate outcomes (N = 1,988)

| **Outcome dimension** | **Standardized β for PARS-3** | **SE** | **t** | **P** | **95% CI for β** | **Adjusted R²** |
| --- | --- | --- | --- | --- | --- | --- |
| Spiritual growth | 0.262 | 0.022 | 12.196 | <0.001 | 0.220 to 0.304 | 0.139 |
| Health responsibility | 0.297 | 0.021 | 13.828 | <0.001 | 0.255 to 0.339 | 0.141 |
| Physical activity | 0.279 | 0.021 | 12.974 | <0.001 | 0.237 to 0.321 | 0.139 |
| Nutrition | 0.295 | 0.021 | 13.749 | <0.001 | 0.253 to 0.337 | 0.142 |
| Interpersonal relations | 0.256 | 0.021 | 11.928 | <0.001 | 0.214 to 0.298 | 0.145 |
| Stress management | 0.271 | 0.021 | 12.672 | <0.001 | 0.229 to 0.313 | 0.147 |

Note: Each model was adjusted for ethnicity, sex, age group, educational level, marital status, employment status, and smoking/drinking history.

Supplementary sensitivity analysis using the non-exercise HPLP-IIR score as the outcome

To directly address the potential conceptual overlap between PARS-3 and the exercise dimension embedded in the HPLP-IIR, we recalculated the HPLP-IIR score after excluding the exercise dimension. The non-exercise HPLP-IIR score was calculated as the HPLP-IIR total score minus the HPLP-IIR exercise dimension score. The main correlation, adjusted regression, and serial indirect association analyses were then repeated using the non-exercise HPLP-IIR score as the outcome variable.

After excluding the exercise dimension, PARS-3 remained significantly correlated with the non-exercise HPLP-IIR score (r = 0.201, P < 0.001). In the adjusted regression model, PARS-3, WHO-5, and SEMCD-6 were all significantly and positively associated with the non-exercise HPLP-IIR score. The serial indirect association model also remained significant, with all bootstrapped confidence intervals excluding zero. These findings indicate that the main association between PARS-3 and HPLP-IIR was not solely attributable to shared exercise-related content.

Supplementary Table S3. Sensitivity analysis using the non-exercise HPLP-IIR score as the outcome

| **Analysis** | **Estimate** | **SE / Boot SE** | **t** | **P** | **95% CI / Boot 95% CI** |
| --- | --- | --- | --- | --- | --- |
| Correlation: PA with non-exercise HPLP-IIR | r = 0.201 | — | — | <0.001 | — |
| Regression: PA | β = 0.196 | — | 9.578 | <0.001 | — |
| Regression: SWB | β = 0.204 | — | 9.775 | <0.001 | — |
| Regression: SEMCD | β = 0.228 | — | 11.073 | <0.001 | — |
| PA → SWB → non-exercise HPLP-IIR | 0.070 | 0.008 | — | — | 0.055 to 0.086 |
| PA → SEMCD → non-exercise HPLP-IIR | 0.053 | 0.008 | — | — | 0.039 to 0.069 |
| PA → SWB → SEMCD → non-exercise HPLP-IIR | 0.024 | 0.003 | — | — | 0.018 to 0.031 |
| Total indirect association | 0.147 | 0.012 | — | — | 0.125 to 0.170 |
| Total association | 0.471 | 0.040 | — | — | 0.391 to 0.545 |

Note: The non-exercise HPLP-IIR score was calculated as the HPLP-IIR total score minus the HPLP-IIR exercise dimension score. Regression and serial indirect association models were adjusted for ethnicity, sex, age group, educational level, marital status, employment status, and smoking/drinking history. PA, SWB, and SEMCD were operationalized using PARS-3, WHO-5, and SEMCD-6 scores, respectively.
